# Consequences of the COVID-19 pandemic on the continuum of care in a cohort of people living with HIV followed in a single center of Northern Italy

**DOI:** 10.1186/s12981-020-00314-y

**Published:** 2020-10-04

**Authors:** Eugenia Quiros-Roldan, Paola Magro, Canio Carriero, Annacarla Chiesa, Issa El Hamad, Elena Tratta, Raffaella Fazio, Beatrice Formenti, Francesco Castelli

**Affiliations:** 1 Department of Infectious Diseases, University of Brescia, Brescia, Italy; 2 Division of Infectious Diseases, ASST Spedali Civili, Brescia, Italy; 3 Central Pharmacy, ASST Spedali Civili, Brescia, Italy

**Keywords:** HIV continuum of care, COVID-19, Public health, SARS-CoV-2, Follow-up, Adherence

## Abstract

**Introduction:**

During the COVID-19 pandemic, hospitals faced increasing pressure, where people living with HIV risked to either acquire SARS-CoV-2 and to interrupt the HIV continuum of care.

**Methods:**

This is a retrospective, observational study. We compared the numbers of medical visits performed, antiretroviral drugs dispensed and the number of new HIV diagnosis and of hospitalizations in a cohort of people living with HIV (PLWH) followed by the Spedali Civili of Brescia between the bimester of the COVID-19 pandemic peak and the bimester of October–November 2019. Data were retrieved from administrative files and from paper and electronic clinical charts. Categorical variables were described using frequencies and percentages, while continuous variables were described using mean, median, and interquartile range (IQR) values. Means for continuous variables were compared using Student’s t-tests and the Mann–Whitney test. Proportions for categorical variables were compared using the *χ*^2^ test.

**Results:**

As of December 31st, 2019, a total of 3875 PLWH were followed in our clinic. Mean age was 51.4 ± 13 years old, where 28% were females and 18.8% non-Italian. Overall, 98.9% were on ART (n = 3834), 93% were viro-suppressed. A total of 1217 and 1162 patients had their visit scheduled at our out-patient HIV clinic during the two bimesters of 2019 and 2020, respectively. Comparing the two periods, we observed a raise of missed visits from 5 to 8% (*p* < 0.01), a reduction in the number of new HIV diagnosis from 6.4 in 2019 to 2.5 per month in 2020 (*p *= 0.01), a drop in ART dispensation and an increase of hospitalized HIV patients due to COVID-19*.* ART regimens including protease inhibitors (PIs) had a smaller average drop than ART not including PIs (16.6 vs 21.6%, *p* < 0.05). Whether this may be due to the perception of a possible efficacy of PIs on COVID19 is not known.

**Conclusions:**

Our experience highlights the importance of a resilient healthcare system and the need to implement new strategies in order to guarantee the continuum of HIV care even in the context of emergency.

## Introduction

Coronavirus disease 2019 (COVID-19) has spread rapidly around the world since the first reports from Wuhan city in China at the end of 2019, and the outbreak was declared as pandemic by WHO on March 12, 2020 [1]. On February 21st, 2020, the first person was reported to be infected with severe acute respiratory syndrome coronavirus 2 (SARS-CoV-2) in Lombardy, in Northern Italy. This region rapidly became the first COVID-19 outbreak in Europe. As of May 2nd, 209.328 COVID-19 cases have been confirmed in Italy, where more than one out of three cases (36.7%) were diagnosed in Lombardy, the most populated Region of Italy [2]. Since the end of February, hospitals were rapidly overcrowded by COVID-19 patients, and both infectious and non-infectious diseases specialists were reassigned to the new COVID-19 wards to cope with the astonishing number of hospitalized COVID-19 cases. Therefore, most hospitals became COVID-19 hotspots, thus potentially putting outpatients with chronic diseases at risk for nosocomial acquisition of SARS-CoV-2. In addition, hospital personnel faced enormous challenges because of staff reduction due to reassignment to COVID-19 care units and COVID-19 quarantining as a consequence of SARS-CoV-2 infection.

The “90–90–90” strategy promoted by UNAIDS is calling for a scaling up of HIV cascade of care to 90% of people living with HIV (PLHIV) diagnosed, 90% linked to care and antiretroviral therapy (ART) and 90% of those patients that adhere to treatment achieving stable viral suppression [3]. In the period between March and April 2020, SARS-CoV-2 hit hard on the province of Brescia, Lombardy, which became the third province in Italy for the number of COVID-19 cases [4]. The Spedali Civili of Brescia is the hub hospital for Infectious Diseases in the whole Brescia province, and the only Hospital dedicated to HIV care. During the pandemic peak, the majority of the working force specialized on infectious diseases in our hospital focused on the cure of COVID-19 patients, putting several barriers and challenges to the HIV care continuum. We normally follow almost 4000 PLWH [5]. The onset of the COVID-19 pandemic in our city made urgent and mandatory to institute an emergency response aimed to maintain HIV care delivery, adapting our out-patients clinic to the new and dramatic changes imposed by the pandemic.

Here we describe the continuum of HIV care in our patients, the changes made in order to guarantee medical assistance during the worst 2 months of the COVID-19 epidemic and the consequences of the pandemic on our patients in this critical period.

## Methods

### Study setting

The present retrospective, observational study was conducted at the University Department of Infectious and Tropical Diseases of the Azienda Socio-Sanitaria Territoriale (ASST) Spedali Civili of Brescia, Lombardy, Northern Italy. The Spedali Civili General Hospital of Brescia is one of the largest hospitals in Italy, and it is also the reference hospital for the School of Medicine of the University of Brescia. It represents the only tertiary referral center for Infectious Diseases and HIV care of the whole Brescia province (about 1.2 million inhabitants).

### Study population

All PLWH aged 18 years and more followed at our out-patients clinic were included.

From the administrative files, we collected data on (i) new HIV diagnosis (ii) number of medical visits in our HIV out-patients clinic (scheduled and then performed through in-person assessment or telemedicine, missed or postponed (iii) dispensation of antiretroviral (ART) medications and (iv) hospitalizations of PLWH. From the electronic and paper clinical records, we retrieved epidemiological and clinical characteristics including sex, origin country, CD4 + T-cells absolute count, HIV RNA load, antiretroviral therapy (ART), diagnosis of admittance if hospitalized.

**Fig. 1:**
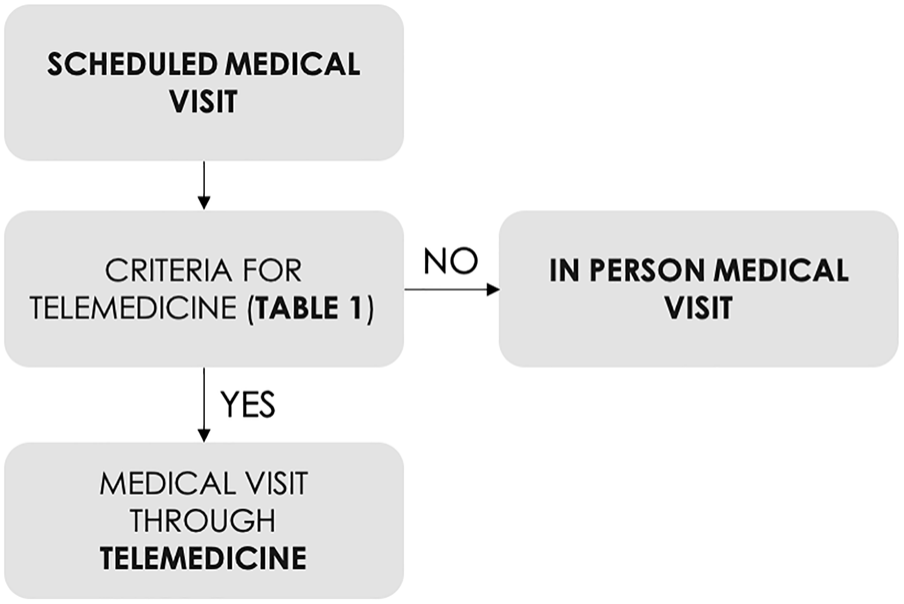
Algorithm for the decision of medical visit modality during the COVID-19 pandemic

Normally, chronic HIV-positive patients with stable and effective ART (HIV RNA < 20 cp/ml for at least 6 months) and CD4 + T-cells > 500 cell/μl are evaluated through blood exams and subsequent medical visit every 6 months at our clinic. Naïve patients, as well as patients with more complicated medical issues which require a tighter follow-up, are seen more often [6]. Antiretroviral drugs are dispensed behind medical prescription every 3 months by our pharmacy, inside the hospital. During the COVID-19 epidemic, we modified the normal HIV management as explained in Fig. 1. Criteria for telemedicine are reported in Table 1.

**Table 1:** Criteria for the decision of the medical visit form

Whether one or more of these criteria was met, the patient was evaluated through a normal medical visit If none of these criteria was present, the visit was performed through telemedicine	CD4 + T-cells < 200 cell/μl and/or < 14%
	HIV RNA > 20 cp/ml in the previous examination
	Switch to a new ART regimen in the last 6 months
	Decrease of CD4 + T-cells > 25% and/or percentage decrease > 10% in the two previous examinations
	Ongoing treatment with direct anti-viral agents (DAAs) for HCV eradication
	Diagnosis of HIV infection or first contact at our clinic within the last 6 months
	Elevation of liver function tests (LFTs) more than twice the normal values
	Liver cirrhosis
	Unexplained decrease of hemoglobin at last exams
	Chronic kidney disease with creatinine clearance < 60 ml/min or decrease of > 25% in creatinine clearance since the last examination
	Clinical history reporting frailty conditions or high risk of loss at follow-up (e.g. psychiatric comorbidities, active drug addiction, oncologic or hematologic comorbidities; pregnancy; kidney transplants; nursing home residents, patients recently released from prison and/or on house arrest)
	Recent new diagnosis and/or admittance in hospital and/or changes in the therapies for comorbidities
	Previous lost at follow-up since more than one year
	Recent diagnosis of HCV infection, HBV infection, Syphilis new infection or reinfection
	Missed answer to telemedicine call

The study comprises two periods: one before and one during the pandemic. In order to describe the changes in the continuum of care with COVID-19 pandemic, we compared the medical visits and the dispensation of ART medications in PLWH occurred between October 1st and November 30th, 2019 and March 1st and April 30th, 2020. We chose October and November 2019 for comparison, because they had similar amounts of festivities, same ARTs available and similar administrative management. At last, we compared the number of new HIV diagnosis and the number of PLWH admitted at our hospital during the COVID-19 pandemic and the mean number of those occurred during the year 2019.

### Statistical analysis

Categorical variables were described using frequencies and percentages, while continuous variables were described using mean, median, and interquartile range (IQR) values. Means for continuous variables were compared using independent group Student’s t-tests when the data were normally distributed and the Mann–Whitney test when they were not. Proportions for categorical variables were compared using the *χ*^2^ test, although Fisher’s exact test was used when the data were sparse.

### Ethics

This study was conducted accordingly to the Declaration of Helsinky and the principles of Good Clinical Practices (GCP). As this study had a retrospective design and was based on routinely collected data, patients informed consent was not required according to the Italian Law (Italian Guidelines for classification and conduction of observational studies, established by the Italian Drug Agency, “Agenzia Italiana del Farmaco – AIFA” on March 20, 2008). For this study a general consent form of the Italian Guarantor was signed by the participants, authorizing the use of retrospective demographical and clinical data, which have been treated according to the present laws.

## Results

As of December 31st, 2019, a total of 3875 PLWH (mean age 51.4 ± 13 years old) were actively followed in our clinic. Of these, 28% were females and 18.8% non-Italian. Overall, 98.9% were on ART (n = 3834), of which 32.1% were assuming an ART regimen that included a protease inhibitor. HIV RNA was < 20 cp/ml in 93% of patients and CD4 +  > 350 cells/mm^3^ in 88.3%.

### New HIV diagnosis

During 2019, a total of 77 new HIV diagnosis were registered at our center, for a monthly mean of 6.4 new diagnosis. About 20% of all new diagnosis were in females. In the bimester of the COVID-19 pandemic we observed only five patients with a first HIV diagnosis (mean 2.5 new diagnosis per month, *p* = 0.01). Of these, only one occurred in March, while the remaining four were made in April. Three out of five new patients were females (60%), and only one of them was not-Italian (20%). AIDS-defining conditions were present at the moment of HIV diagnosis in two patients (one had Kaposi sarcoma and the second one a non-Hodgkin lymphoma). Among the three remaining, two received HIV diagnosis during routine screenings for pregnancy and the last one in the context of a hospitalization for enteric intussusception. The median CD4 + T-cell count at diagnosis was 236 cell/mm^3^ (range 12–479 cell/mm^3^).

### Number of medical visits at our HIV out-patients clinic

**Fig. 2:**
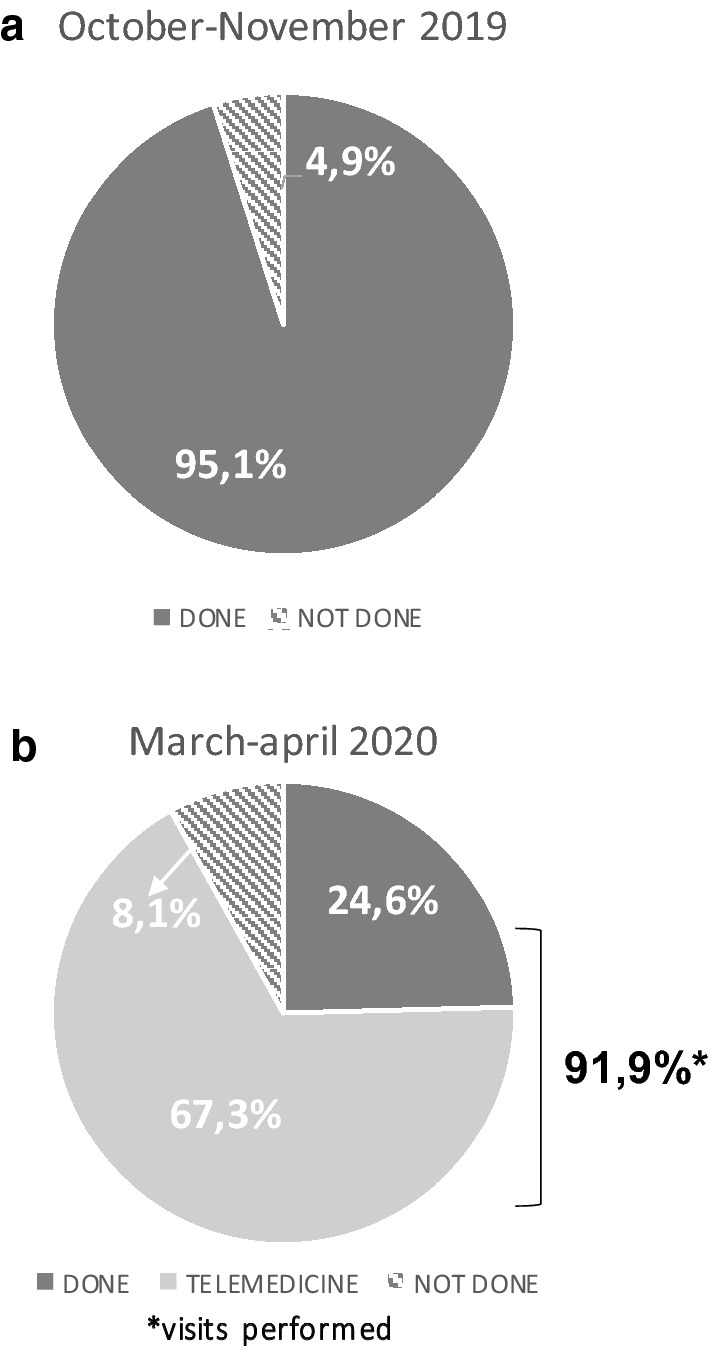
a Overall view of the percentage of patients that performed their scheduled medical visit in the first bimester**; **b Overall view of the percentage of patients that performed their scheduled medical visit (both through telemedicine and through in-person evaluation) in the second bimester

During the bimesters October–November 2019 and March–April 2020 a total of 1217 and 1162 patients had their visit scheduled at our out-patient HIV clinic, respectively. The overall view of the performed visits is shown in Fig. 2 and Additional file 1. Thanks to the telemedicine program initiated at our clinic at the beginning of the COVID-19 pandemic, a total of 782 (67.3%) patients resulted eligible for this modality and performed their visit through this new useful tool in March and April 2020 (Fig. 2).

**Fig. 3:**
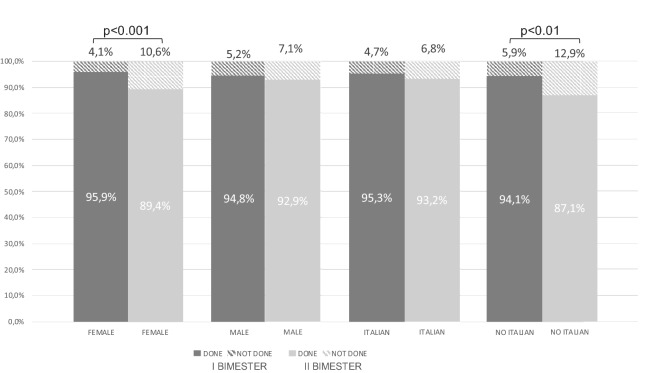
Proportion of patients that performed the scheduled medical visit in the first bimester in comparison with the second bimester according to sex and nationality

Missing visits were significantly higher in the second bimester (8.1 vs 4.9%; *p* < 0.01). As shown in Fig. 3, females tended to skip their scheduled appointment during the pandemic period more than normally (10.6 vs 4.1%,* p* < 0.001).

Not Italian patients showed a similar behavior and were more likely to avoid medical visits in the second bimester (12.9 vs 5,9%, *p* < 0.01). Males registered a trend of decreased presence at visit, even though not statistically significant (5.2 vs 7%, *p* = 0.055) as well as Italian patients (5.2% vs 6.8%, *p* = 0.07).

### Dispensation of antiretroviral medications

We have found that the number of patients to whom ART was dispensed during March and April 2020 decreased of− 23,1% in comparison with October–November 2019 (Table 2). The highest decrease was observed in non-Italian compared to Italian (− 27.4 vs − 20.1%, *p* < 0.05) and in females in comparison with males (− 23.6 vs -20.5%, NS). When we compared March and April separately with the mean of patients to whom ART was dispensed during the first bimester, we observed a higher drop of drugs dispensation in March (− 33.6%) with a trend to normalization in April (− 12.6%).

**Table 2:** Trends in antiretroviral dispensation to PLWH during the bimester of March–April 2020 and October–November 2019

	Nationality		Sex		Total %
	Italian %	Non-italian %	Males %	Females %	
% Decrease in March–April 2020 compared to October–November 2019*	− 20.1	− 27.4*	− 20.5	− 23.6	− 23.1
% Decrease in March 2020 compared to the mean of October–November 2019*	− 30.0	− 30.6	− 29.1	− 32.7	− 33.6
% Decrease in April 2020 compared to the mean of October–November 2019*	− 10.3	− 24.1**	− 11.9	− 14.4	− 12.9

The percentage was calculated as the difference in the overall number of patients that collected ART between the different study periods

*p < 0.05

**p < 0.01

Lastly, we explored potential differences among the number of drug packs dispensed in the two bimesters. We compared the number of drug packs dispensed in March–April 2020 and the mean of packs dispensed between June and December 2019. To this end, we chose to analyze the trends of the most used single tablet regimens (STRs) in our center, including an integrase inhibitor (lamivudine/abacavir/dolutegravir) and a protease inhibitor (PIs) (darunavir/cobicistat/tenofovir alafenamide/emtricitabine) (data not shown). Both STRs registered an average drop of − 21.6 and − 16.6% (p < 0.05) respectively for the 2020 bimester in comparison with the second semester of 2019.

### Number of PLWH hospitalized

During 2019, a total of 92 PLWH were admitted in our ward (7.7 patients/month). Between March and April 2020, 25 PLWH were admitted to our Infectious Diseases clinic (12.5 patients/months). Among these, 12 patients were hospitalized because of SARS-CoV-2 infection (48%).

Epidemiological, clinical and therapeutic characteristics of HIV-positive patients admitted for COVID-19 can be summarized as follow.

The average age was 56 years (IQR, 54–58; range, 52–63 years) and seven (58%) were males. All patients were assuming ART regularly and were virologically suppressed at admission. One patient was prescribed with a regimen including a protease-inhibitor (darunavir-boosted cobicistat). The average number of CD4 + T-cells was 527 cell/mm^3^ (IQR, 361–665 cell/mm^3^, range 265–886 cell/mm^3^). The average length of hospitalization was 12 days (IQR, 7–15). Hypertension (n = 2, 17%), cardiovascular (n = 2, 17%) and oncologic diseases (n = 4, 33%) and diabetes (n = 2, 17%) were the most common coexisting conditions. The great majority of patients reported fever at symptoms onset (n = 9, 75%), dry cough (n = 7, 58%) and dyspnea (n = 3, 25%). At hospital admission, the average SO_2_/FiO_2_ was 416 (IQR, 425–462; range 111–476). Most of patients (92%) were classified as moderate COVID-19 cases (SO_2_/FiO_2_ > 300 mmHg and radiological findings of pneumonia) and only one as severe (SO_2_/FiO_2_ ≤ 300 mmHg with radiological pneumonia). Overall, 83% of patients received hydroxychloroquine for COVID-19 treatment (n = 10), where seven out of twelve patients (58%) maintained their usual ART during the hospitalization period. None of them died, while one patient required to be transferred to the intensive care unit for critical conditions.

## Discussion

The first 2 months after the pandemic started have produced important changes in the management of PLWH in our HIV care unit. The main goals during the COVID-19 pandemic have been to both prevent PLWH and health care professionals from SARS-CoV-2 infection and to maintain an appropriate HIV continuum of care.

Our findings highlight several issues. The first UNAIDS target (90% of all people living with HIV should know their HIV status) [3] is based on HIV testing and is the first step towards initiation into the HIV care continuum. Our clinic receives patients from a large province with more than 1 million inhabitants, where the incidence of HIV infection is estimated to be one of the highest in Italy, 5.8/100,000 compared to 4.7/100,000 in the whole country [7]. From a public health perspective, the reduction of the occurrence of new cases is detrimental in order to reduce viral circulation. Promoting HIV testing with early diagnosis represents the essential entry point for both treatment and prevention efforts. Here we show as the COVID-19 pandemic had a negative impact on HIV screening programs in our province. Indeed, we observed a drop in the number of new HIV cases in comparison with the monthly mean of new HIV diagnosis occurred in the 2019 (2.5 vs 6.4 new HIV cases/month, *p* = 0.01). On March 8th, 2020 the Italian government declared the implementation of community containment measures in order to contain the diffusion of SARS-CoV-2 [8], which included social distancing, movement restrictions and quarantine for certain or suspected cases. Predictably, these measures drastically reduced the access to routine HIV testing [9, 10]. For a near future, it will be important to organize health services in order to guarantee a safe and continuous access to HIV testing, which is a fundamental health service and a detrimental step towards HIV elimination. Females and non-Italian patients resulted to be less adherent to follow-up visits during the pandemic period. This data needs to be further investigated and addressed. Anyway, we believe that it is of fundamental importance to identify the more at-risk populations as auspicated by Hargreaves et al. [11], in order to better address their needs and behaviors.

The second target [3], (90% of all people with HIV diagnosis should be maintained linked to HIV care) is based in offering and maintaining HIV care in all patients. Soon, hospital visits may be restricted again because of future implementations of city lockdowns or traffic controls. From the beginning of March, we adopted the telemedicine tool with the purpose of avoiding the interruption of the continuum of HIV care. We performed structured phone interviews, where the physician, together with collecting information about clinical status and ART adherence, also shared laboratory results and future appointments with PLWH. Telemedical consultations have helped to solve the otherwise unmanageable discrepancy between the *#stayathome* strategy and the need of a continuous medical assistance as also Ridgway et al. experienced [12]. Our preliminary experience shows that a large proportion of patients (67.3%) is eligible for a telemedical appointment. In the next years, we may think of this strategy for semesterly evaluation of stable patients. Moreover, this new tool may be helpful to avoid stigma related to be seen in an Infectious Diseases clinic, to decrease the loss of hours of work for medical visits and to reduce the illness perception. Nonetheless, we recommend that more complex cases keep on being considered for in-person medical evaluation and more often than every 6 months. Furthermore, for a more comprehensive understanding of the efficacy of this approach and its impact on HIV care, we will need to evaluate HIV plasmatic suppression in the months to come.

The last target [3], (90% of PLWH with suppressed viral plasmatic loads) is based in assuring antiretroviral therapy to PLWH and in reinforcing adherence to ART. According to Italian regulations, PLWH need to collect their ART at the hospital pharmacy either personally or through a person authorized with a written approval form. Immediately after the beginning of the outbreak, many local charities began to offer home delivery services for ART medications through volunteers, in order to avoid patients exposure to SARS-CoV-2. Initially, this service was not homogenous, relying on local associations or individual initiatives (e.g. municipal police, veterans, local charities, local Red Cross committee, etc.), while at the end of March, this service was coordinated on a national basis, and supported by Red Cross volunteers. In fact, we observed that although the mean decrease during the study period was − 23% compared to 2019, in March the decrease reached − 33,6%, when in April this trend tended to normalize (− 12,6%). Once again, we will need to wait to better understand the consequences of this period on ART adherence and HIV replication.

Because many PLWH contacted us asking whether protease inhibitors would have been protective against SARS-CoV-2 infection [13–16], we analyzed the changes on the collection of STRs including protease inhibitors (darunavir/cobicistat/tenofovir alafenamide/emtricitabine) and STRs not including PIs (lamivudine/abacavir/dolutegravir) at our pharmacy. What we observed is that STRs including PIs had a smaller average drop than STR including integrase inhibitors (16.6 vs 21.6%, *p* < 0.05). Although we cannot assert whether patients on PIs have been more compliant to ART collection because of the possible effect of these drugs on COVID-19, we think that this data is interesting for it to be pointed out.

Lastly, we described the clinical consequences of SARS-CoV-2 infection in our cohort. We recorded almost a doubling in the number of hospitalizations in PLWH in comparison with 2019, with a mean of 12.5 patients per month in the second bimester in comparison with 7.6 patients per month in the first. This increase can be ascribed to SARS-CoV-2 infection in PLWH: indeed, on average, we recorded six HIV/SARS-CoV-2 coinfections per month in 2020. Overall, we managed few cases of HIV/SARS-CoV-2 coinfections. Most of them were admitted with a moderate COVID-19 disease and had optimal outcomes, despite a higher average age and a higher proportion of patients with comorbidities (75%) in comparison with other cohorts [17, 18]. Besides, all patients, except three, had CD4 + T-cells > 350/mm^3^, indicating no severe immune deficiency. As more data were coming out for the efficacy of PIs against SARS-CoV-2 in vivo [19], half of our patients maintained their usual ART and did not switch to a PI during the time of hospitalization. Of those hospitalized, only one patient was prescribed chronically with darunavir/cobicistat and was virosuppressed at admittance. Our observation, although very modest, may add evidence for a lack of a protective role of darunavir against SARS-CoV-2 [20], while more robust evidences are awaited [21]. At last, our experience does not support the idea of an excess of morbidity and mortality among PLWH with viral suppression affected by COVID-19 pneumonia. Moreover, we agree with the recent suggestion from Jones et al.[22] of not modifying ART in order to attempt to treat SARS-CoV-2 infection.

Our study has important limitations. First, this is a retrospective study without follow-up, therefore it is not possible to ascertain the consequences and the efficacy of our strategies on the last 90 of our strategy (90% of all people receiving antiretroviral therapy with viral suppression). Moreover, the number of hospitalized PLWH may be too small to offer a comprehensive understanding of COVID-19 disease in PLWH. About this, we may have “lost” some HIV/SARS-CoV-2 coinfections, due to eventual hospitalizations in hospitals other than ours. It must be said anyway, that HIV patients normally tend to be centralized and hospitalized in our ward.

Strength of this study is to have analyzed the main falls in the continuum of HIV care in our area during the emergency period. Now, we have learnt that telemedicine and home delivery services for ART medications are useful and powerful tools. Anyway, our data also emphasize the need to detect the populations at high risk for HIV care attrition (in our case, females and non-Italian patients) and to implement effective retention tools focused on these. We fell in maintaining high levels of routine HIV screening, which is crucial for early diagnosis and for reduction of viral circulation. Therefore, we learned that we have to keep on offering valid alternatives for HIV screening, where several health-care services and other community-based organizations were closed in this period due to the COVID-19 emergency. Moreover, in-person evaluations need to be maintained for those cases which are considered clinically more complicated or more at risk of loss.

## Conclusions

Even though we cannot have definitive data in such a short period, our preliminary impressions are that telemedicine can be a useful tool, which can make communications with some group of patients who are already engaged on the continuum of care available and easier. This strategy may be extremely useful during pandemic times, but also beyond them and may be further implemented, for example through video calls. HIV testing services and strategies need to be supported and promoted even in critical periods, in the near future. Females and non-Italian patients are an especially at risk population for loss at follow-up, at least in the short period, so that special attention should be paid to these population in order to make them feel safe and to let them understand the fundamental importance of a continuous adherence to ART even during emergency periods. Unfortunately, we may say, it seems that we need to adapt our HIV care services to emergency settings. We hope that the strategy that we found will result effective in the near future, and that our experience may help to open a debate on the best ways to manage the HIV-positive population in this new reality.

## Supplementary information

**Additional file 1.** Number of medical visits expected, performed through in person evaluation or telemedicine and not performed at our clinic during the two bimesters.
