# Supplementary material for: Consequences of the COVID-19 pandemic on the continuum of care in a cohort of people living with HIV followed in a single center of Northern Italy
Source: AIDS Res Ther. 2020 Oct 4;17:59. doi: 10.1186/s12981-020-00314-y (PMC7533114; doi:10.1186/s12981-020-00314-y)
Supplement: Supplementary file 1 — Additional file 1. Number of medical visits expected, performed through in person evaluation or telemedicine and not performed at our clinic during the two bimesters. [file 12981_2020_314_MOESM1_ESM.docx]

**Supplementary material 1.** Number of medical visits expected, performed through in person evaluation or telemedicine and not performed at our clinic during the two bimesters.

|  | **October (n=667)** | | **Total** | **November (n=550)** | | **Total** | **Total I bimester** | **March (n=665)** | | **Total** | **April (n=507)** | | **Total** | **Total II bimester** |
| --- | --- | --- | --- | --- | --- | --- | --- | --- | --- | --- | --- | --- | --- | --- |
|  | **Done** | **Not Done** |  | **Done** | **Not Done** |  |  | **Done** | **Not Done** |  | **Done** | **Not Done** |  |  |
| Females | 176 (96,2%) | 7 (3.8%) | 183 (27,4%) | 149 (95,5%) | 7 (4,5%) | 156 (28,4%) | 339 (27,9%) | 166 (88,3%) | 22 (11,7%) | 188 (28,7%) | 129 (90,8%) | 13 (9,2%) | 142 (28%) | 330 (89,4%) |
| Males | 459 (94,8%) | 25 (5,2%) | 484  (72,6%) | 373 (94,7%) | 21 (5,3%) | 394 (71,6%) | 878 (72,1%) | 428 (91,6%) | 39 (8,4%) | 467 (71,3%) | 345 (94,5%) | 20 (5,5%) | 365 (72%) | 832 (92,9%) |
| **Total** | 635 (95,2%) | 32 (4,8%) | **667** | 522 (94,9%) | 28 (5,1%) | **550** | **1217** | 594 (90,7%) | 61 (9,3%) | **655** | 474 (93,5%) | 33 (6,5%) | **507** | **1162** |
| Italian | 524 (95,3%) | 26 (4,7%) | 550 (82,5%) | 441 (95,2%) | 22 (4,8%) | 463 (84,2%) | 1013 (83,2%) | 479 (91,8%) | 43 (8,2%) | 522 (79,7%) | 380 (95%) | 20 (5%) | 400 (78,9%) | 922 (79,3%) |
| Not-Italian | 111 (94,9%) | 6 (5,1%) | 117 (17,5%) | 81 (93,1%) | 6 (6,9%) | 87 (15,8%) | 204 (16,8%) | 115 (86,5%) | 18 (13,5%) | 133 (20,3%) | 94 (87,9%) | 13 (12,1%) | 107 (21,1%) | 240 (20,7%) |
| **Total** | 635 (95,2%) | 32 (4,8%) | **667** | 522 (94,9%) | 28 (5,1%) | **550** | **1217** | 594 (90,7%) | 61 (9,3%) | **655** | 474 (93,5%) | 33 (6,5%) | **507** | **1162** |
